# Genetic Diversity of Carbapenem-Resistant *Enterobacteriaceae* (CRE) Clinical Isolates From a Tertiary Hospital in Eastern China

**DOI:** 10.3389/fmicb.2018.03341

**Published:** 2019-01-15

**Authors:** Minhui Miao, Huiyan Wen, Ping Xu, Siqiang Niu, Jingnan Lv, Xiaofang Xie, José R. Mediavilla, Yi-Wei Tang, Barry N. Kreiswirth, Xia Zhang, Haifang Zhang, Hong Du, Liang Chen

**Affiliations:** ^1^Department of Clinical Laboratory, The Second Affiliated Hospital of Soochow University, Suzhou, China; ^2^Department of Clinical Laboratory, Jiangyin Hospital of Traditional Chinese Medicine, Jiangyin, China; ^3^Department of Clinical Laboratory, The Fifth People’s Hospital of Suzhou, Suzhou, China; ^4^Department of Laboratory Medicine, The First Affiliated Hospital of Chongqing Medical University, Chongqing, China; ^5^Public Health Research Institute Tuberculosis Center, New Jersey Medical School, Rutgers University, Newark, NJ, United States; ^6^Memorial Sloan Kettering Cancer Center, New York, NY, United States; ^7^Department of Clinical Laboratory, The North District of Affiliated Suzhou Hospital, Nanjing Medical University, Suzhou, China

**Keywords:** carbapenem-resistant *Enterobacteriaceae*, carbapenemase, resistance mechanism, genetic diversity, plasmid

## Abstract

The prevalence of carbapenem-resistant *Enterobacteriaceae* (CRE) is increasing globally, with different molecular mechanisms described. Here we studied the molecular mechanisms of carbapenem resistance, including clonal and plasmid dissemination, of 67 CRE isolates collected between 2012 and 2016 from a tertiary hospital in Eastern China, an CRE endemic region. Species identification and susceptibility testing were performed using the BD Phoenix Automated Microbiology System. Isolates were characterized by PCR (for carbapenemases, ESBLs, AmpC and porin genes), multilocus sequence typing (MLST), pulsed-field gel electrophoresis (PFGE), and conjugation transfer experiments. Selected *bla*_KPC-2_ -harboring plasmids were subjected to next-generation sequencing using the Illumina Miseq platform. Among the 67 CRE isolates, 42 *Klebsiella pneumoniae*, 10 *Serratia marcescens*, 6 *Enterobacter cloacae*, 2 *Raoultella ornithinolytica*, 2 *K. oxytoca*, 1 *K. aerogenes*, and 4 *Escherichia coli* isolates were identified. Six different carbapenemases were detected, including *bla*_KPC-2_ (*n* = 45), *bla*_KPC-3_ (*n* = 1), *bla*_NDM-1_ (*n* = 6), *bla*_NDM-5_ (*n* = 1), *bla*_IMP-4_ (*n* = 2), and *bla*_VIM-1_ (*n* = 2); *bla*_OXA-48_-like genes were not detected. One *E. cloacae* strain possessed both *bla*_NDM-1_ and *bla*_KPC-3_, while two *E. cloacae* isolates harbored *bla*_NDM-1_ and *bla*_VIM-1_. ESBLs (CTX-M, SHV, and TEM) and/or AmpC (CMY, DHA, and ACT/MIR) genes were also identified in 59 isolates, including 13 strains that lacked carbapenemases. Several insertions or stop codon mutations were found within porin genes of *K. pneumoniae, E. coli* and *S. marcescens* isolates, both with and without carbapenemases. The 42 *K. pneumoniae* isolates belonged to 12 different sequence types (ST), with ST11 being the most common, while the 6 *E. cloacae* isolates comprised 4 different STs. The 10 *S. marcescens* all shared the same PFGE pulsotype, suggestive of clonal spread. Complete plasmid sequencing and PCR screening revealed both intra-strain and inter-species spread of a common *bla*_KPC-2_-harboring plasmid in our hospital. Taken together, our study revealed extensive genetic diversity among CRE isolates form a single Chinese hospital. CRE isolates circulating in the hospital differ significantly in their species, STs, porin genes, carbapenemase genes, and their plasmid content, highlighting the complex dissemination of CRE in this endemic region.

## Introduction

*Enterobacteriaceae* are among the most common pathogenic Gram-negative bacteria (GNB), causing various community- and healthcare-acquired infections. Nowadays, multidrug resistant GNB (MDR-GNB) are increasingly described in clinical settings, and carbapenems are regarded as the most effective antibiotic therapy for infections caused by MDR-GNB. However, as a result of clinical use of carbapenems since the late 1980s, the occurrence of carbapenem-resistant *Enterobacteriaceae* (CRE) has been increasingly reported worldwide, including in China (Gupta et al., 2011; Nordmann and Poirel, 2014). According to reports by the China Antimicrobial Resistance Surveillance System (CARSS), the detection rate of CRE in China increased from 2005 to 2014, demonstrating a continuous upward trend and suggesting a worsening situation (Hu et al., 2016).

Carbapenem resistance in *Enterobacteriaceae* can arise through distinct molecular mechanisms, mainly via the production of carbapenemases, but also as a consequence of outer membrane porin dysfunction coupled with hyper-production of AmpC cephalosporinases or extended-spectrum β-lactamases (ESBLs) (Bush and Jacoby, 2010; Bush and Fisher, 2011). Carbapenemases are a group of β-lactamases that are capable of hydrolyzing carbapenem antibiotics, in addition to cephalosporins and other β-lactam antimicrobials. Three major class of carbapenemases are widespread globally in clinical CRE isolates, including class A (mainly KPC), class B (VIM, NDM, and IMP) and class D (OXA-48 and its variants, OXA-162 and OXA-181, etc.) (Ambler, 1980; Hall et al., 2003; Nordmann and Poirel, 2014). Notably, carbapenemase genes are primarily carried by large conjugative plasmids, thereby facilitating horizontal transfer of carbapenem resistance among different bacterial strains and species. As mentioned above, another common mechanism of carbapenem resistance involves the combination of porin dysfunction with hyper-production of AmpC (e.g., CMY, DHA, and ACT) or ESBLs (e.g., TEM, SHV, and CTX-M) (Logan and Weinstein, 2017). The lack of the production of porins can preclude diffusion of antibiotics through bacterial membranes, along with the action of ESBL and AmpC enzymes, thereby producing the phenotype of carbapenem resistance in *Enterobacteriaceae* (Paterson and Bonomo, 2005; Jacoby, 2009; Bush and Jacoby, 2010). Unlike carbapenemases, porin dysfunction-associated resistance is not able to spread through horizontal transfer, but may disseminate via clonal expansion.

Dissemination of CRE usually demonstrates geographical and temporal variation in specific global regions. Here we characterized CRE clinical strains collected from a tertiary hospital in eastern China between August 2012 and August 2016. The genetic relatedness, antimicrobial susceptibility, and carbapenem-resistance mechanisms of these CRE isolates were examined in detail.

## Materials and Methods

### Identification of Carbapenem-Resistant *Enterobacteriaceae* Isolates

Sixty-seven unique (one isolate per patient) CRE clinical isolates were retrospectively collected from the Second Affiliated Hospital of Soochow University between August 2012 and August 2016. In this study, carbapenem resistance was defined as resistance to meropenem or imipenem based on 2016 Clinical and Laboratory Standards Institute (CLSI) guidelines (CLSI, 2016). The isolates were collected from various sources, including sputum (*n* = 48), urine (*n* = 14), blood (*n* = 2), catheter (*n* = 1), ascites (*n* = 1), and drainage fluid (*n* = 1). Species identification was performed using the Phoenix 100 Automated Microbiology System (Becton-Dickinson, United States), and confirmed by 16S rRNA sequencing (Weisburg et al., 1991). This study was approved by the institutional review board (IRB) of The Second Affiliated Hospital of Soochow University. The clinical isolates were retrospectively collected, and patient data were not included in this study.

### Antimicrobial Susceptibility Testing

The minimal inhibitory concentrations (MICs) of the CRE strains were performed using the Phoenix 100 Automated Microbiology System and interpreted according to CLSI criteria (CLSI, 2016). A total of 18 antibiotics belonging to eight classes of antimicrobials were tested, including carbapenems (imipenem and meropenem), penicillins (ampicillin), β-lactam/β-lactamase inhibitor complexes (amoxicillin-clavulanate, ampicillin-sulbactam, and piperacillin-tazobactam), cephalosporins (cefazolin, cefuroxime, ceftazidime, and cefepime), monocyclic β-lactams (aztreonam), aminoglycosides (gentamicin and amikacin), fluoroquinolones (ciprofloxacin and levofloxacin), folate metabolic pathway inhibitors (trimethoprim-sulfamethoxazole), colistin and tigecycline.

### Detection of Carbapenemases, ESBLs, AmpC, and Porin Genes

Polymerase chain reaction (PCR) was performed to investigate the presence of carbapenemase-encoding genes, including *bla*_KPC_, *bla*_NDM_, *bla*_VIM_, *bla*_IMP_ and *bla*_OXA-48_. Simultaneously, we examined ESBLs (CTX-M, SHV, and TEM), AmpC cephalosporinases (CMY, ACT, and DHA), and mutation in porin encoding genes (OmpK35/OmpF, OmpK36/OmpC), using PCR followed by Sanger sequencing. Oligonucleotide primers used for screening the above genes have been reported previously (Mammeri et al., 2010; Bokaeian et al., 2015; Candan and Aksoz, 2015; Sugawara et al., 2016).

### Multilocus Sequence Typing (MLST)

Multilocus sequence typing (MLST) was conducted to investigate the genetic relationships of different CRE isolates. PCR followed by Sanger sequencing was used to detect conserved housekeeping genes in distinct species, including *Klebsiella* spp. (*gapA, infB, mdh, pgi, phoE, rpoB*, and *tonB*), *E. coli* (*adk, fumC, gyrB, icd, mdh, purA, and recA*), and *E. cloacae* (*dnaA, fusA, gyrB, leuS, pyrG, rplB, and rpoB*). Allelic profiles and sequence types (STs) were determined according to species-specific MLST databases^1^ Primers used for MLST were described in previous reports (Diancourt et al., 2005; Wirth et al., 2006; Miyoshi-Akiyama et al., 2013; Herzog et al., 2014).

### Pulsed-Field Gel Electrophoresis (PFGE)

Raoultella *ornithinolytica* and Serratia *marcescens* strains (for which no MLST schemes Are available) Were further investigated by PFGE using a CHEF Mapper Power Module instrument (Bio-Rad, United States). In brief, genomic DNA Was digested With *Xba* I, and then electrophoresed Under the following conditions: voltage 6 V/cm, running time 18–19 h, temperature 14°C, and pulse times of 5–40 s (*R. ornithinolytica*) and 5–20 s (*S. marcescens*). *Salmonella* strain H9812 Was used as a control strain and size marker. Clonal relatedness Between strains Was evaluated based on the criteria proposed by Tenover et al. (1995).

### Plasmid Sequencing and Screening

Conjugation transfer experiments were performed with selected *bla*_KPC-2_-harboring strains and rifampicin-resistant *E. coli* EC600. Experiments were carried out using mixed broth culture method as described previously (Chen et al., 2014a). Transconjugants were identified by detecting resistance genes using PCR. Plasmid DNA from *E. coli* EC600 transconjugants harboring single plasmids was extracted using a Qiagen Plasmid Midi Kit (Qiagen, Valencia, CA, United States), and sequenced using the Illumina Miseq system (Illumina, United States) (Du et al., 2016). Sequencing reads were assembled *de novo* into contigs using SPAdes (Bankevich et al., 2012), then manually inspected using Geneious 9.1^2^; and gaps were closed by PCR and Sanger sequencing.

**FIGURE 1:**
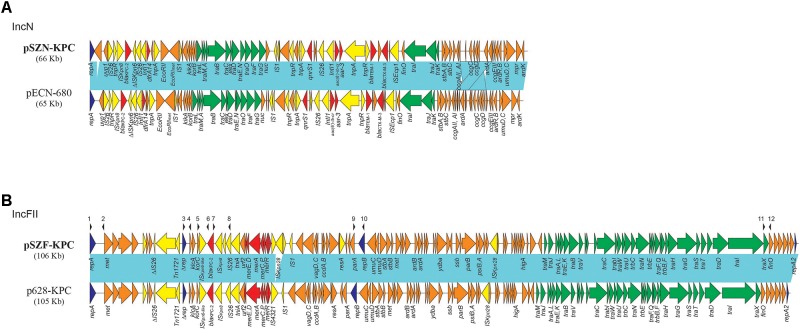
Comparative analysis of **(A)** IncN and **(B)** IncFII *bla*_KPC-2_–like harboring plasmids. Light blue shading denotes shared regions of homology with >99% identities. ORFs are portrayed by arrows and colored according to predicted gene function: orange arrows indicate plasmid scaffold regions; green arrows denote genes associated with the *tra* locus; dark blue arrows indicate replication-associated genes; Red arrows denote antimicrobial and mercury resistance genes; and yellow arrows indicate accessory genes. Small black arrowheads above the plasmids indicate the locations of primers used for PCR screening (primer sequences are shown in Table 1).

A PCR mapping strategy was developed to detect a common *bla*_KPC-2_-harboring pSZF_KPC/p628-KPC-like plasmid sequenced in this study. The scheme includes six individual PCR reactions. PCR-I was designed to target the region spanning IncFII replicon gene *repA* and its downstream DNA methylase gene, while PCR-V was designed to target the junction of second replicon gene *repB* and its upstream *parA*. PCR-II, III, and PCR-IV were designed to target the *bla*_KPC-2_ neighboring regions of *Δrep*-*klcA*, *klcA*-*bla*_KPC-2_, and *bla*_KPC-2_-IS*26*, respectively (Figure 1). PCR-VI was used to detect the *traX*-*finO* junction in pSZF_KPC/p628-KPC-like plasmids. The oligonucleotide primer target regions for identification of pSZF_KPC/p628-KPC-like plasmids are shown in Figure 1, and primer sequences are listed in Table 1.

**Table 1:** Oligonucleotide primers used to screen pSZF_KPC/p628-KPC-like plasmids.

PCRs	No.*^a^*	Name	Sequences	Size (bps)	Targets
PCR-I	1	repA-F1	GGGAACAACTACACGCGACT	1447	Junction between IncFII *repA* and DNA methylase gene
	2	repA-R1	GTTTTGCCCATGCTCAACTT		
PCR-II	3	Δrep-F	TGAGACAAGTCCCTCCCCTA	1138	Junction between *Δrep* and *klcA*
	4	klcA-R	GCCCTTTCATTTGCTGGTAA		
PCR-III	5	korC-F	GGTGAGCAAAACCAACCCTA	1417	Junction between *korC* and *bla*_KPC-2_
	6	KPC-R	ACAAGGATGACAAGCACAGC		
PCR-IV	7	KPC-F	CGAGTTTAGCGAATGGTTCC	2030	Junction between *bla*_KPC-2_ and downstream IS*26*
	8	IS26-R	CGCCTGGTAAGCAGAGTTTT		
PCR-V	9	parA-F	GCCCAGTGACATCAGATACG	870	Junction between *parA* and *repB*
	10	repB-R	TAAACTGGCCCTCAAGCAGT		
PCR-VI	11	traX-F	CCAGGTGTCGTTTATGCTCA	563	Junction between *traX* and *finO*
	12	finO-R	GGTTTTCGTTTCAGGCTCAG		


*^a^The primer locations are illustrated in Figure 1.*

## Results

### Species and Antimicrobial Susceptibility

A total of 67 non-duplicate CRE isolates were collected from our hospital from August 2012 to August 2016, consisting of 42 *K. pneumoniae*, 2 *K. oxytoca*, 1 *K. aerogenes*, 10 *S. marcescens*, 6 *E. cloacae*, 2 *R. ornithinolytica,* and 4 *E. coli*. The results of antimicrobial susceptibility testing are shown in Table 2. All isolates were resistant to ampicillin, cefazolin, cefuroxime, imipenem, meropenem, and amoxicillin/clavulanate, and exhibited high resistance rates to most of the other β-lactam antibiotics tested. The most active compounds against all isolates were colistin (97.0% susceptible), tigecycline (94.0% susceptible), trimethoprim/sulfamethoxazole (56.7% susceptible) and amikacin (50.7% susceptible).

**Table 2:** Susceptibility of CRE isolates against different antimicrobial agents.

Antimicrobial agents^∗^	All isolates (*n* = 67)	*Klebsiella pneumonia* (*n* = 42)	*Klebsiella oxytoca* (*n* = 2)	*Enterobacter cloacae* (*n* = 6)	*Enterobacter coli* (*n* = 4)	*Klebsiella aerogenes* (*n* = 1)	*Raoultella ornithinolytica* (*n* = 2)	*Serratia marcescens* (*n* = 10)
	
	*n* (%)	*n* (%)	*n* (%)	*n* (%)	*n* (%)	*n* (%)	*n* (%)	*n* (%)
AMP	0 (0)	0 (0)	0 (0)	0 (0)	0 (0)	0 (0)	0 (0)	0 (0)
CZO	0 (0)	0 (0)	0 (0)	0 (0)	0 (0)	0 (0)	0 (0)	0 (0)
CXM	0 (0)	0 (0)	0 (0)	0 (0)	0 (0)	0 (0)	0 (0)	0 (0)
CAZ	4 (6)	1 (2.4)	0 (0)	1 (16.7)	0 (0)	0 (0)	1 (50)	0 (0)
FEP	3 (4.5)	2 (4.8)	0 (0)	0 (0)	0 (0)	0 (0)	1 (50)	0 (0)
AMC	0 (0)	0 (0)	0 (0)	0 (0)	0 (0)	0 (0)	0 (0)	0 (0)
SAM	1 (1.5)	1 (2.4)	0 (0)	0 (0)	0 (0)	0 (0)	0 (0)	0 (0)
TZP	4 (6)	1 (2.4)	1 (50)	1 (16.7)	0 (0)	1 (100)	0 (0)	0 (0)
ATM	5 (7.5)	1 (2.4)	2 (100)	1 (16.7)	1 (25)	0 (0)	0 (0)	0 (0)
IPM	0 (0)	0 (0)	0 (0)	0 (0)	0 (0)	0 (0)	0 (0)	0 (0)
MEM	0 (0)	0 (0)	0 (0)	0 (0)	0 (0)	0 (0)	0 (0)	0 (0)
GEN	10 (15.0)	5 (11.9)	1 (50)	1 (16.7)	1 (25)	0 (0)	2 (100)	0 (0)
AMK	34 (50.7)	15 (35.7)	2 (100)	3 (50.0)	2 (50)	1 (100)	1 (50)	10 (100)
CIP	6 (9.0)	4 (9.5)	0 (0)	1 (16.7)	0 (0)	0 (0)	1 (50)	0 (0)
LEV	11 (16.4)	7 (16.7)	1 (50)	1 (16.7)	0 (0)	0 (0)	2 (100)	0 (0)
SXT	38 (56.7)	23 (54.8)	2 (100)	2 (33.3)	1 (25)	1 (100)	0 (0)	9 (90)
TGC	63 (94.0)	38 (90.5)	2 (100)	6 (100)	4 (100)	1 (100)	2 (100)	10 (100)
CL	65 (97.0)	40 (95.2)	2 (100)	6 (100)	4 (100)	1 (100)	2 (100)	10 (100)

*^∗^IPM, imipenem; MEM, meropenem; AMP, ampicillin; AMC, amoxicillin-clavulanate; SAM, ampicillin-sulbactam; TZP, piperacillin-tazobactam; CZO, cefazolin; CXM, cefuroxime; CAZ, ceftazidime; FEP, cefepime; ATM, aztreonam; GEN, gentamicin; AMK, amikacin; CIP, ciprofloxacin; LEV, levofloxacin; SXT, trimethoprim-sulfamethoxazole; TGC, tigecycline; CL, colistin. n (%), n = number of isolates that were susceptible; % = percentage of isolates susceptible.*

### Detection of Carbapenemase Genes

In this study, 54 (80.6%) of the 67 CREs were found to harbor at least one carbapenemase gene (Table 3). However, the distribution of carbapenemases among different species varied significantly, while the frequencies of carbapenemase-producing *Enterobacteriaceae* (CPE) in different species were 100%, 88.1%, 50.0%, and 50.0% in *E. coli*, *K. pneumoniae*, *E. cloacae* and *S. marcescens*, respectively.

**Table 3:** Molecular characteristics of CRE clinical isolates.

Species	Number	Carbapenemases (n, %)	ESBLs and AmpC (n, %)^∗^	Mutations of encoding Porin (n, %)^∗^	STs (n, %)
*K. pneumoniae*	42	KPC-2 (37, 88.1%)	CTX-M-9 (6, 14.3%), CTX-M-14 (7, 16.7%), CTX-M-65 (19, 45.2%), SHV-12 (26, 61.9%), DHA-1 (21, 50.0%)	*ompK35* (26, 61.9%), *ompK36* (26, 61.9%)	ST11 (25, 59.5%), ST774 (3, 7.1%), ST1107 (3, 7.1%), ST12 (2, 4.8%), ST45 (2, 4.8%), ST8 (1, 2.4%), ST36 (1, 2.4%), ST211 (1, 2.4%), ST218 (1, 2.4%), ST395 (1, 2.4%), ST655 (1, 2.4%), ST697 (1, 2.4%)
*K. oxytoca*	2	NDM-1 (1, 50%), IMP-4 (1, 50%)	-	N/A	ST135 (1, 50%), ST180 (1, 50%)
*K. aerogenes*	1	KPC-2 (1, 100%)	-	N/A	N/A
*R. ornithinolytica*	2	KPC-2 (1, 50%), IMP-4 (1, 50%)	CTX-M-15 (1, 50%), SHV-12 (1, 50%)	N/A	N/A
*S. marcescens*	10	KPC-2 (5, 50%)	CTX-M-14 (10, 100%)	*ompF* (10, 100%)	N/A
*E. coli*	4	KPC-2 (1, 25%), NDM-1 (2, 50%), NDM-5 (1, 25%)	CTX-M-15 (2, 50%), SHV-12 (1, 25%), CMY-2 (2, 50%)	*ompF* (1, 25%)	ST167 (1, 25%), ST1488 (1, 25%), ST3234 (1, 25%), ST354 (1, 25%)
*E. cloacae*	6	KPC-3 (1, 16.7%), NDM-1 (3, 50%), VIM-1 (2, 33.3%)	ACT (6, 100%), CTX-M-3 (3, 50%), CTX-M-9 (1, 16.7%), CTX-M-14 (2, 33.3%), SHV-12 (2, 33.3%)	*ompF* (1, 16.7%)	ST231 (3, 50%), ST120 (1,16.7%), ST97 (1, 16.7%), ST421 (1, 16.7%)

*^∗^-, negative; N/A, not available or not performed.*

Various carbapenemases were identified among the 54 CPE isolates, including *bla*_KPC-2_ (*n* = 45), *bla*_KPC-3_ (*n* = 1), *bla*_NDM-1_ (*n* = 6), *bla*_NDM-5_ (*n* = 1), *bla*_IMP-4_ (*n* = 2), and *bla*_VIM-1_ (*n* = 2). No strains were found to carry *bla*_OXA-48_-like genes. Among these, KPC was the most predominant carbapenemase (80.7%), and was primarily found in *K. pneumoniae* (37/42, 88.1%). Three *E. cloacae* strains were found to co-harbor two carbapenemase-encoding genes, with one strain harboring both *bla*_KPC-3_ and *bla*_NDM-1_, while the other two harboring both *bla*_NDM-1_ and *bla*_VIM-1_.

### Other Mechanisms Associated With Carbapenem Resistance

As described above, PCR failed to identify any carbapenemases among 13 out of 67 CRE strains, including 5 *K. pneumoniae*, 3 *E. cloacae* and 5 *S. marcescens*, suggesting that other mechanisms may have contributed to the phenotypic carbapenem resistance among these isolates. We therefore examined ESBL-encoding genes (*bla*_TEM_, *bla*_SHV_, and *bla*_CTX-M_), AmpC-encoding genes (*bla*_CMY_, *bla*_DHA_, and *bla*_ACT_) and outer membrane porin genes. ESBL and AmpC genes were tested in all isolates, while outer membrane porin genes were examined in *K. pneumoniae* (*ompK35* and *ompK36*), as well as in *E. coli*, *E. cloacae*, and *S. marcescens* (*ompF* and *ompC*).

Fifty-nine isolates were found to carry at least one ESBL and/or AmpC gene, including 13 non-carbapenemase-producing strains. Specifically, 51 (76.1%) strains were found to carry *bla*_CTX-M_ genes, including *bla*_CTX-M-65_ (*n* = 19), *bla*_CTX-M-14_ (*n* = 19), *bla*_CTX-M-9_ (*n* = 7), *bla*_CTX-M-15_ (*n* = 3), and *bla*_CTX-M-3_ (*n* = 3). These were found in several species, including *K. pneumoniae* (*n* = 32), *S. marcescens* (*n* = 10), *E. cloacae* (*n* = 6), *E. coli* (*n* = 2), and *R. ornighinolytica* (*n* = 1). In addition, 30 strains were positive for *bla*_SHV-12_, most of which were *K. pneumoniae* (26/30, 86.7%), while 21 *K. pneumoniae* harbored *bla*_DHA-1_, 6 *E. cloacae* possessed *bla*_ACT_, and two *E. coli* were positive for *bla*_CMY-2_.

Outer membrane porin gene sequence analysis showed that 27 *K. pneumoniae* isolates harbored *ompK35* and/or *ompK36* mutations. *ompK35* mutations (*n* = 26) were exclusively due to premature stop codons, while *ompK36* mutations included glycine-aspartic acid (GD) insertions at amino acid positions 134–135 (*n* = 23), IS*10* insertions (*n* = 2), and stop codons (*n* = 1). Five non-carbapenemase-producing *K. pneumoniae* CRE contained at least one outer membrane porin gene mutant (*ompK35* or *ompK36*) while also harboring ESBL genes *bla*_CTX-M_ or *bla*_SHV-12_, which likely explains the carbapenem resistance among these isolates. Sequence analysis of *ompF* and *ompC* genes in *S. marcescens* showed that they all possess mutated *ompF*, with premature stop codons at amino acid position 72. One non-carbapenemase-producing *E. cloacae* isolate was also found to carry an *ompF* mutation (stop codon), in addition to *bla*_ACT_ and *bla*_CTX-M-9_. However, two non-carbapenemase-producing *E. cloaca*e CRE isolates did not display mutations in either *ompF* or *ompC*, although they were found to harbor ESBL genes (either *bla*_CTX-M-14_ or *bla*_CTX-M-3_). We suspect additional mechanisms, such as efflux pumps or penicillin-binding protein modifications, may contribute to carbapenem resistance in the latter two isolates. Therefore, carbapenem resistance among 11 out of 13 non-carbapenemase-producing CRE isolates may be explained by the combination of porin gene mutants and the presence of ESBL or AmpC-encoding genes, while the resistance mechanisms in two non-carbapenemase-producing *E. cloaca*e CRE isolates remain to be determined. Meanwhile, among the 54 CPE isolates, 28 (51.9%) also carry at least one outer membrane porin mutant.

### Distribution of MLST Sequence Types and PFGE Patterns

Multilocus sequence typing results showed that 42 *K. pneumoniae* belonged to 12 different STs, with ST11 being the most common (25/42, 59.5%). All ST11 isolates possessed the same OmpK35 stop codon, while 23 of them harbored the 134–135 GD OmpK36 mutant. The two *K. oxytoca* isolates belonged to ST135 and ST180, while the 4 *E. coli* strains were assigned to ST1488, ST3234, ST167, and ST354. The six *E. cloacae* also comprised 4 STs, including ST231 (*n* = 3), ST120 (*n* = 1), ST421 (*n* = 1), and ST97 (*n* = 1). Two *R. ornithinolytica* and 10 *S. marcescens* strains were further analyzed by *Xba* I-PFGE. The results showed that all 10 *S. marcescens* strains shared the same PFGE pattern, suggesting clonal spread (Supplementary Figure 1). In addition, the ten *S. marcescens* isolates were collected within 7 months in 2013 from three closed wards (respiratory, neurology, and renal wards) and mostly from respiratory samples (*n* = 9), suggestive of the likelihood of a small *S. marcescens* outbreak. As aforementioned, all *S. marcescens* harbored the same OmpF mutant, and among them, 5 were also found to carry *bla*_KPC-2_. By contrast, the two *R. ornithinolytica* isolates displayed unrelated PFGE patterns (data not shown).

### Sequencing and Screening of *bla*_KPC-2_-Harboring Plasmids

Since KPC-2 is the most common carbapenemase found in our hospital, we sought to determine the complete sequences of *bla*_KPC-2_ -harboring plasmids spreading through our institution. We selected 4 *bla*_KPC-2_-harboring strains, including two *K. pneumoniae* (ST8 and ST11), one *E. coli* (ST3234), and one *S. marcescens* for plasmid conjugation and complete plasmid sequencing.

The *bla*_KPC-2_-harboring plasmid (subsequently named pSZN_KPC) isolated from *K. pneumoniae* ST8 belonged to incompatibility group N. Plasmid pSZN_KPC is 65,604 bp in length, with an average G+C content of 53.3 %, and contains 88 predicted open reading frames (ORFs) (Figure 1). A BLAST search of the pSZN_KPC plasmid sequence against the GenBank database^3^ showed that pSZN_KPC exhibits a high degree of identity to the previously published IncN plasmid pECN580 (accession no. KF914891) from an *E. coli* strain found in China (Chen et al., 2014a), with 100% query coverage and overall 99% nucleotide identity. The major difference is that the region harboring antirestriction protein gene *ardA* in plasmid pSZN_KPC was located downstream of gene *ccgCD* (Figure 1), likely as a consequence of recombination.

The *bla*_KPC-2_-harboring plasmids from *K. pneumoniae* ST11 strain Kp715, *E. coli* ST3234 strain Ec732, and *S. marcescens* strain Sm703 were highly similar (each differed by only 4 SNPs), and belonged to the same IncFII incompatibility group. This plasmid (subsequently named pSZF_KPC) is 106,201 bp in length with a G+C content of 53.2 %, and harbors 122 predicted ORFs. Comparative sequence analysis for plasmid pSZF_KPC showed that it is highly similar (100% query coverage and >99% nucleotide identity) to p628-KPC (accession no. KP987218) from a *K. pneumoniae* isolate collected in our hospital in 2010 (Wang et al., 2015). Compared to p628-KPC, the main difference in pSZF_KPC is that there is an additional IS*Kpn18* element, with IS*4321* located downstream of the *mcr* operon (Figure 1). The sequences of pSZN_KPC and pSZF_KPC have been deposited in GenBank under the accession numbers MH917122 and MH917123, respectively.

The plasmid sequence results described above suggest that pSZF_KPC/p628-KPC-like plasmids have been spreading in our hospital since at least 2010, undergoing horizontal transmission into different species. We therefore developed a set of PCR assays to screen for the presence of pSZF_KPC/p628-KPC-like plasmids among our 67 CRE isolates. The PCR results showed that 27 (64.2%) of the 42 *bla*_KPC-2_ positive strains harbored pSZF_KPC/p628-KPC-like plasmids. The 27 strains encompassed different species and STs, including *K. pneumoniae* ST11 (*n* = 14), ST774 (*n* = 3), ST1107 (*n* = 2), ST211 (*n* = 1), ST655 (*n* = 1), and ST218 (*n* = 1); *S. marcescens* (*n* = 4); and *E. coli* ST3234 (*n* = 1). These pSZF_KPC/p628-KPC-like plasmid-harboring isolates were collected in 8 different wards, spanning from 2012 to 2016, indicating the frequent horizontal transfer of this common plasmid among *Enterobacteriaceae* in our hospital.

## Discussion

At the present time, KPC, NDM and OXA-48 are the most common carbapenemases worldwide (Nordmann and Poirel, 2014; Sugawara et al., 2016). KPCs are most frequently identified in *K. pneumoniae* from the United States, China, Colombia, Israel, Greece, and Italy, while NDMs are primarily found in *K. pneumoniae*, *E. coli* and *Enterobacter spp.* from the Indian subcontinent, and OXA-48-like carbapenemases in *K. pneumoniae* and *E. coli* from North Africa and Turkey (Nordmann and Poirel, 2014). In addition, the spread of CPEs has been associated with several high-risk clones. One notable example involves the global spread of KPCs, which has been largely associated with *K. pneumoniae* clonal group 258 (CG258) strains, of which ST258 is the most predominant KPC-producing *K. pneumoniae* clone in North America, while ST11 is most common in East Asia, especially China (Patel and Bonomo, 2013; Chen et al., 2014b; Zhang et al., 2017).

China, in particular eastern China, is regarded as one of the primary global endemic regions for CRE (Zhang et al., 2017). In this study, we phenotypically and genetically characterized the CRE isolates collected from an eastern Chinese hospital, and investigated the molecular mechanisms underlying carbapenem resistance. Our study revealed several interesting findings.

Firstly, the CRE isolates were recovered from seven different *Enterobacteriaceae* species. Although carbapenem resistance has been frequently identified in *K. pneumoniae*, *Enterobacter* spp., and *E. coli*, it is fairly uncommon in other species such as *R. ornithinolytica* and *S. marcescens*. *R. ornithinolytica* is mostly recovered from the environment and rarely causes severe infections in humans; nevertheless several reports have described the emergence of carbapenem-resistant *R. ornithinolytica* in China (Zhou et al., 2013; Qin et al., 2014; Yang et al., 2018). Notably, in this study we identified two carbapenem-resistant *R. ornithinolytica*, harboring *bla*_KPC-2_ and *bla*_IMP-4_, respectively, suggesting that different carbapenemase plasmids have spread into *R. ornithinolytica.* In contrast, carbapenem resistance in *S. marcescens* has been historically associated with a specific group of carbapenemases, SMEs. However, in this study, none of the ten *S. marcescens* strains were found to carry *bla*_SME_, and the observed carbapenem resistance was likely due to the mutations of OmpK35 porin encoding genes, as well as production of CTX-M-14 and KPC-2. Interestingly, only five of the ten *S. marcescens* strains were found to harbor *bla*_KPC-2_, although PFGE results suggested that the spread of carbapenem-resistant *S. marcescens* was largely clonal. We suspect it is likely that the *S. marcescens* OmpK35 mutant further acquired a *bla*_KPC-2_ plasmid (e.g., pSZF_KPC/p628-KPC-like).

In this study, *K. pneumoniae* was the most common CRE species, accounting for 62.7% (42/67) of all CRE isolates. Among these, ST11, a member of the epidemic CG258 clone, was the predominant ST (59.5%, 25/42), which is consistent with the molecular epidemiology described in other regions of China (Zhou et al., 2013; Zhang et al., 2017; Yang et al., 2018). However, in our study carbapenem-resistant *K. pneumoniae* were found in 12 different STs, including some STs rarely associated with carbapenem resistance (e.g., ST774 and ST1107). Our plasmid screening results showed that the diversity of STs was largely due to the frequent transfer of a common *bla*_KPC-2_ vector into different *K. pneumoniae* genetic backgrounds.

Secondly, our study revealed diverse molecular mechanisms of carbapenem resistance within our hospital, albeit with carbapenemase production the primary cause. Diverse types of carbapenemases, including KPC-2, KPC-3, NDM-1, NDM-5, IMP-4, and VIM-1, were identified, whereas in most Chinese hospitals, usually only KPC and/or NDM carbapenemases are prevalent (Hu et al., 2014). It is noteworthy that four different classes of carbapenemases were found in our hospital, suggesting that different carbapenemase producing plasmids/strains are spreading locally. Notably, however, ∼20% strains were non-carbapenemase producers. It is therefore likely that besides carbapenemases, other mechanisms such as porins defects and production of ESBLs (TEM, SHV, and CTX-M) or AmpC β-lactamases (CMY, DHA, and ACT) significantly contributed to carbapenem resistance in our hospital. Meanwhile, it is worth noting that 51.9% (28/54) of carbapenemase-producing isolates also harbored additional porin gene mutations, potentially rendering antimicrobial treatment more challenging in comparison to strains without porin mutations (Clancy et al., 2013).

Lastly, we identified a widespread *bla*_KPC-2_-harboring plasmid vector within our institution. Several pSZF_KPC/p628-KPC-like plasmids were identified within distinct *K. pneumoniae* clones (STs), as well as in different species (*K. pneumoniae*, *S. marcescens* and *E. coli*). Our PCR-based screening of *bla*_KPC-2_-positive *K. pneumoniae* isolates revealed that this plasmid is widely disseminated in our hospital and was found in nearly two third (22/37) of KPC-2 positive isolates, highlighting the importance of plasmid horizontal transfer in the dissemination of KPC. More importantly, this plasmid was identified in different *K. pneumoniae* STs, as well as other species, suggesting that intra-strain and inter-species transfer of this plasmid have significantly contributed to the spread of carbapenem resistance in our hospital.

In conclusion, our study revealed extensive genetic diversity among CRE isolates from a single Chinese hospital. Both clonal expansion (e.g., *K. pneumoniae* ST11 and *S. marcescens*) and plasmid horizontal transfer (i.e., pSZF_KPC/p628-KPC-like plasmids) were identified. Different carbapenemases classes and outer membrane porin defects were found in several species. CRE isolates circulating in our hospital differ significantly in their species, STs, porin genes, carbapenemase genes, and plasmid content, highlighting complex dissemination of CRE within our hospital. Further studies are required to understand the factors underlying the genetic diversity of CRE in our hospital, in order to effectively control further spread of carbapenem resistance.

## Author Contributions

MM, HW, and PX contributed to work, data analysis, and manuscript preparation. SN, JL, JM, Y-WT, and BK prepared the manuscript. XX contributed to work and data analysis. XZ and HZ analyzed the data. HD and LC contributed to study design, data analysis, and manuscript preparation.

## Conflict of Interest Statement

The authors declare that the research was conducted in the absence of any commercial or financial relationships that could be construed as a potential conflict of interest.

## Footnotes

1 http://pubmlst.org/.

2 http://www.geneious.com/

3 http://blast.ncbi.nlm.nih.gov/Blast.cgi
