# Supplementary figures and images for: Genetic Diversity of Carbapenem-Resistant Enterobacteriaceae (CRE) Clinical Isolates From a Tertiary Hospital in Eastern China
Source: Front Microbiol. 2019 Jan 15;9:3341. doi: 10.3389/fmicb.2018.03341 (PMC6340961; doi:10.3389/fmicb.2018.03341)

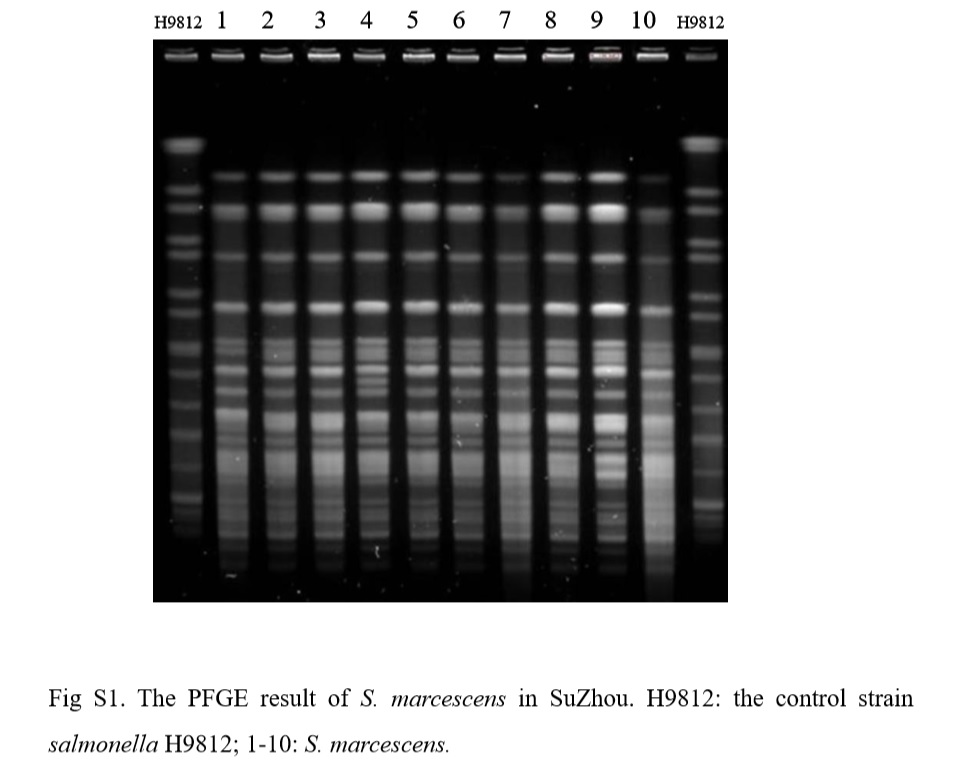

Supplement: Supplementary file 1 [file Image_1.JPEG]
